# Beneficial Effects of Omega-3 Fatty Acids on Obesity and Related Metabolic and Chronic Inflammatory Diseases

**DOI:** 10.3390/nu17071253

**Published:** 2025-04-03

**Authors:** Donia Jerab, Ferdinand Blangero, Paulo César Trindade da Costa, José Luiz de Brito Alves, Rym Kefi, Henda Jamoussi, Beatrice Morio, Assia Eljaafari

**Affiliations:** 1 CarMeN Laboratory, Institut National de Recherche pour l’ Agriculture, l’ Alimentation et l’Environnement, UMR1397, Institut National de la Santé et de la Recherche Médicale, U 1060, Université Claude Bernard Lyon I, 69310 Pierre-Bénite, Francebeatrice.morio@inrae.fr (B.M.); 2 Laboratory of Biomedical Genomics and Oncogenetics, Institut Pasteur de Tunis, Tunis 1002, Tunisia; rym.kefi@pasteur.utm.tn; 3 Department of Nutrition, Health Sciences Center, Federal University of Paraíba, João Pessoa 58051-900, Braziljose.luiz@academico.ufpb.br (J.L.d.B.A.); 4 Research Unit “Obesity: Etiopathology and Treatment, UR18ES01”, Faculty of Medicine, Tunis El Manar University, Tunis 2092, Tunisia; henda.jamoussi@gmail.com; 5 Department of Clinical Research, Hospices Civils de Lyon, 69002 Lyon, France

**Keywords:** omega-3 polyunsaturated fatty acids, obesity, inflammation, Th17 cells, chronic metabolic diseases, psoriasis, rheumatoid arthritis, inflammatory bowel disease, multiple sclerosis

## Abstract

Omega-3 polyunsaturated fatty acids (*n*-3 PUFAs) are known to help resolve inflammation through generation of anti-inflammatory eicosanoids and specialized pro-resolving mediators, including resolvins, protectins, and maresins. Through binding to the GPR120/FFAR4 receptor, their beneficial effects result from phospholipid membrane remodeling, impairment of inflammatory signaling molecules clustering, subsequent inhibition of NF-κB and inflammasome activation, and a reduction in oxidative stress. Obesity, a chronic inflammatory disease that contributes to metabolic disorders, is alleviated by *n*-3 PUFAs. In the adipose tissue (AT) of individuals with obesity, *n*-3 PUFAs counteract hypoxia, inhibit immune cell infiltration and AT inflammation, improve insulin sensitivity, and reduce fat mass. Beyond AT, *n*-3 PUFAs also alleviate other metabolic disorders such as metabolic-associated steatotic liver disease (MASLD), gut dysbiosis, and/or renal dysfunction. In cardiovascular disease (CVD), they are mainly recommended as a secondary prevention for patients with coronary heart disease risks. This review provides an in-depth analysis of the benefits of *n*-3 PUFAs in obesity and related metabolic diseases, examining both the mechanistic and clinical aspects. Additionally, it also explores the effects of *n*-3 PUFAs in obesity-related chronic inflammatory conditions, including inflammatory bowel disease, psoriasis, rheumatoid arthritis, osteoarthritis, and multiple sclerosis, by targeting specific pathophysiological mechanisms. Clinical applications and limitations of *n*-3 PUFAs are discussed based on findings from human clinical trials.

## 1. Introduction

Obesity is a chronic inflammatory disease that often leads to co-morbidities such as type 2 diabetes (T2D), metabolic-associated steatotic liver disease (MASLD), and cardiovascular diseases (CVD) [1]. In 2022, the prevalence of obesity reached 890 million people worldwide, underlining its status as a major public health concern [2]. The onset of obesity is usually driven by sedentary lifestyle and hypercaloric diet, leading to energy imbalance and subsequent metabolic disorders. Excess calories are stored as fat in adipose tissue (AT), leading to hyperplasia, hypertrophy, AT remodeling, homeostasis disruption, and the switch of AT environment from an anti-inflammatory and insulin-sensitive state to a pro-inflammatory, hypoxic, and insulin-resistant state. Following this, ectopic deposition of fatty acids (FAs) in other organs and propagation of inflammation to the periphery contribute to metabolic disorders [3]. Beyond metabolic disorders, obesity-mediated inflammation exacerbates various auto-immune and inflammatory diseases, such as inflammatory bowel diseases (IBD), psoriasis, rheumatic diseases, multiple sclerosis, and cancers, as previously reviewed [4,5].

Current approaches to mitigate obesity focus on rebalancing energy expenditure by increasing physical activity and reducing excess calorie intake. Besides pharmacological [6] and bariatric surgical strategies [7], adjunction of dietary interventions that emphasize the consumption of fish and fruits and vegetables rich in bioactive compounds, such as the Mediterranean diet, is often required to promote better metabolic health [8]. In the Mediterranean diet, dietary components such as polyphenols are known to exert their effects through antioxidant activity, epigenetic modulation, and microbiota-dependent pathways [9]. Dietary fibers act by modulating gut microbiota, producing short-chain fatty acids, and improving gut barrier integrity [10,11], while *n*-3 PUFAs act by directly resolving inflammation, modulating membrane composition, and improving lipid metabolism. In addition to their direct anti-inflammatory properties, *n*-3 PUFAs also exert their effects by competing with *n*-6 PUFAs for enzymatic conversion. Therefore, by inhibiting the synthesis of pro-inflammatory prostaglandins derived from *n*-6 PUFAs, which are abundant in many dietary sources, such as PGE_2_, *n*-3 PUFAs help shift the balance toward a more anti-inflammatory state [12].

Beyond their potent anti- inflammatory and adipogenic effects within adipose tissues [13], as well as their systemic beneficial effects, *n*-3 PUFAs may thus help reduce obesity and related metabolic disorders and chronic inflammatory diseases. The aim of this review is to provide a comprehensive analysis of the benefits of *n*-3 PUFAs with respect to these diseases.

## 2. Results

### 2.1. Beneficial Effects of Omega-3 Fatty Acids on Obesity

#### 2.1.1. ω-3/ω-6 Eicosanoid Generation

FAs are identified by the length of their carbon chain and the number and position of unsaturated sites on carbons. FAs are thus classified as saturated FAs (SFAs, 0 double bonds), monounsaturated fatty acids (MUFAs, 1 double bond), or polyunsaturated fatty acids (PUFAs, ≥2 double bonds). Among PUFAs, *n*-3 and *n*-6 PUFAs differ in the position of the first carbon–carbon double bond relative to the terminal methyl group. Thus, alpha-linolenic acid (ALA) is referred to as an 18:3 *n*-3 PUFA, indicating that it is composed of 18 carbons and three double bonds, with the first unsaturated site occurring at the third carbon from the terminal methyl group. Linoleic acid (LA), which is designated as an 18:2 *n*-6 PUFA, contains 18 carbons and two double bonds, with the first double bond occurring at the sixth carbon from the methyl end group. ALA and its derivatives, eicosapentaenoic acid (EPA, 20:5 *n*-3 PUFA) and docosahexaenoic acid (DHA, 22:6 *n*-3 PUFA), are the predominant *n*-3 PUFAs [14].

**Figure 1:**
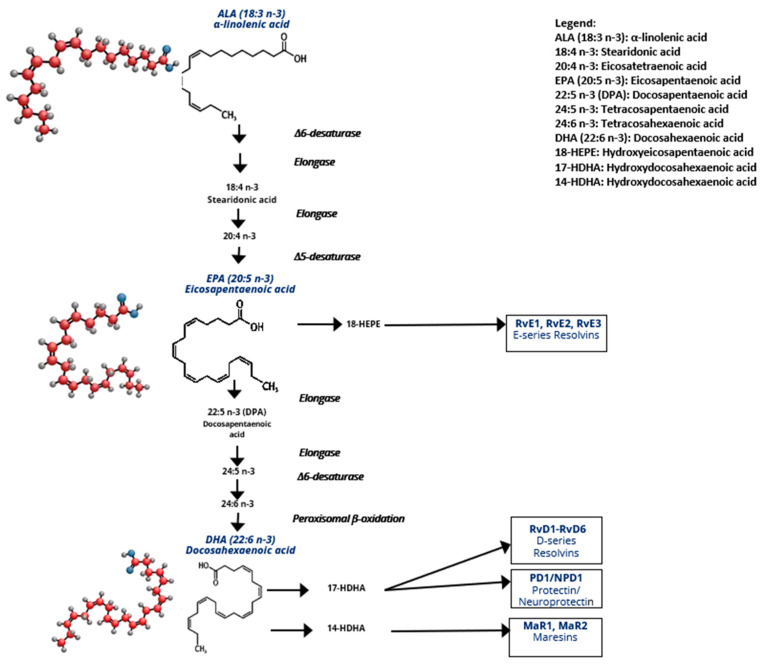
Metabolism of *n*-3 PUFAs. Conversion of ALA into EPA and DHA metabolites and EPA and DHA into resolvins, maresins, and protectins. Abbreviations: HEPE: hydroxyperoxyeicosapentaenoic acid; HDHA: hydroxyperoxydocosahexaenoic acid.

Once incorporated into membranes, LA is metabolized into arachidonic acid (AA 20:4 *n*-6), a key precursor of several pro-inflammatory lipid mediators. In response to stimuli, AA and LA, which are stored in the phospholipid membranes, can be released through the action of phospholipase A2 (PLA2) [15]. In the cytoplasm, AA is metabolized by constitutive cyclooxygenases (COX-1) and inflammation-inducible cyclooxygenase (COX-2), as well as by lipoxygenase (LOX) and other enzymes, into lipid derivatives known as eicosanoids, such as PGD2, PGE2, TXA2, and LTB4 mediators. These eicosanoids are pro-inflammatory and play crucial roles in metabolic and chronic inflammation diseases. By replacing LA or AA within the phospholipids, dietary supplementation with *n*-3 PUFAs reduces the *ω*-6/*ω*-3 ratio within the membranes. When released from membranes, *n*-3 PUFAs also compete with *n*-6 PUFAs for the LOX and COX enzymes involved in eicosanoid synthesis. ALA-derived metabolites such as EPA or DHA are substrates for COX, LOX, and cytochrome P450, which contribute to the synthesis of prostaglandin E3 (PGE3), prostaglandin D3 (PGD3), thromboxane A3 (TXA3), and leukotriene B5 (LTB5), which are less pro-inflammatory than AA-derived mediators [14]. In addition, EPA and DHA are able to be converted into specialized pro-resolving mediators (SPMs) via the COX pathway for resolvins and protectins or the LOX pathway for maresins. Indeed, resolvins D 1–6, protectin 1, and maresins 1–2 are derived from DHA, while resolvins 1–3 are derived from EPA, as shown in Figure 1. All of these PMs are known to contribute to the resolution of inflammation, either by (i) limiting neutrophil infiltration, or trans-endothelial migration of leukocytes, (ii) promoting macrophage phagocytosis, or (iii) inhibiting TNFα and/or IL1β [16,17].

#### 2.1.2. Dietary Sources of *n*-3 PUFAs

ALA is an essential PUFA, but EPA and DHA are considered conditionally essential because their endogenous synthesis from ALA is extremely limited. Therefore, they must be provided by the diet. ALA is mainly obtained from plant sources, such as flaxseeds, rapeseeds, and chia seeds, or from dried fruits such as nuts or walnuts, while EPA and DHA are mainly provided by marine sources, such as oily fish [18]. EPA and DHA can also be generated from ALA but at very low levels, since in humans, the conversion rate of ALA to EPA or DHA is less than 5%, and is further influenced by the *n*-6/*n*-3 PUFA ratio [19]. Thus, a high intake of *n*-6 PUFAs limits the conversion of ALA into EPA or DHA. The recommended ω-6/ω-3 ratio for good health is 5:1. Such a ratio is met and even exceeded in the Japanese population, where the ratio averages 4:1, while this ratio averages 10:1 in Europe and even 20:1 in the United States [20]. Western diets are thus characterized by a high *ω*-6/*ω*-3 ratio, and are associated with increased risks of obesity and metabolic disorders [21]. In the African continent, in particular in Tunisia, a recent cross-sectional study comparing obese patients with and without metabolic syndrome showed that those without metabolic disorders were more likely to adhere to a Mediterranean-style diet, with a higher intake of oily fish [22]. Moreover, in another study, the average ω-6/ω-3 ratio reached 17:1 in the Tunisian T2D population, with none of these patients achieving the recommended dietary intake of *n*-3 PUFAs [23].

Recent dietary guidelines recommend eating at least two servings of fish per week, equivalent to 500 mg of EPA + DHA [24].

#### 2.1.3. Mechanisms of the Anti-Inflammatory Effects of *n*-3 PUFAs

In addition to their ability to compete with *n*-6 PUFAs in the phospholipid membranes for enzymes needed for eicosanoid synthesis, *n*-3 PUFAs may act as agonists for several free FA receptors expressed on a wide range of cells. Notably, EPA and DHA have been shown to activate FFAR4, also known as GPR120, a G protein-coupled receptor [25,26]. This leads to the recruitment of β-arrestin 2 to the plasma membrane. The GPR120/β-arrestin 2 complex is then internalized into the cytoplasmic compartment, where it binds to the TAK1-binding protein (TAB1) and blocks its association with TAK1. This results in the inhibition of the IKK-β/NF-κB and JNK/AP-1 signaling pathways and a reduction in TNF-α and IL-6 transcription [27]. B-arrestin2 has also been found to act downstream of GPR120 and GPR40 to inhibit NLRP3 inflammasome activation, which results in the inhibition of caspase-1 activation and a reduction in the subsequent secretion of IL-1β and IL-18 pro-inflammatory cytokines. *n*-3 PUFA supplementation in high-fat diet (HFD) mice has thus been demonstrated to prevent NLRP3 inflammasome-dependent insulin resistance in vivo [28].

Another mechanism by which *n*-3 PUFA may inhibit AT inflammation is through their ability to bind to peroxisome proliferator-activated receptor (PPAR) nuclear receptors, particularly PPAR-α and PPAR-γ. Once activated by ligand binding, these PPAR isoforms form heterodimers with the retinoid X receptor (RXR) and bind to peroxisome proliferator-activated receptor response elements (PPREs) to trans-repress the activity of transcription factors such as NF-κB. PPAR-α and PPAR-γ are also involved in the regulation of lipid metabolism and adipocyte differentiation, [29,30]. *n*-3 PUFAs have thus been shown to modulate inflammation in adipocytes and immune cells, improve adipogenesis and lipogenesis, and enhance insulin sensitivity through binding to PPAR-α and PPAR-γ [31,32,33].

*n*-3 PUFA action may also result from their incorporation into plasma membranes at the expense of *n*-6 PUFAs, leading to the disruption of lipid rafts. Lipid rafts are regions rich in cholesterol, phospholipids, and sphingolipids that form rigid membrane domains able to anchor receptors and signaling molecules, including those involved in the immunological synapse. Incorporation of *n*-3 PUFAs into these rafts disrupts their structure, thereby inhibiting immune response [34]. Lipid rafts are also involved in the dimerization of toll like receptor 4 (TLR-4) and recruitment of MYD88, leading to NF-κB activation and subsequent inflammation [35]. Of interest, in obesity, reduced intestinal barrier integrity due to gut dysbiosis leads to lipopolysaccharide (LPS) release and endotoxemia [36]. Inflammation may then occur via the binding of LPS to TLR4. However, disruption of lipid rafts by *n*-3 PUFAs reduces the translocation of TLR4 and myeloid differentiation primary response (MYD88) to the membrane, and thus inhibits NF-kB activation [32,35]. A study by Bidu et al. supports the role of *n*-3 PUFAs in reducing gut dysbiosis and inflammation. Using the Fat-1 model in which mice constitutively produce ALA this study demonstrated that mice did not develop gut dysbiosis and displayed reduced metabolic endotoxemia and AT inflammation when fed an obesogenic diet. Moreover, they also demonstrated that transplantation of their gut microbiota to wild-type mice prevented obesity and glucose intolerance, therefore suggesting that *n*-3 PUFAs may also reduce fat mass and insulin resistance by modulating the gut microbiota [37].

#### 2.1.4. Beneficial Effects of *n*-3 PUFAs on AT Inflammation and Adipose Metabolism in Obesity

In addition to their anti-inflammatory properties, *n*-3 PUFAs may improve metabolic dysfunction. Indeed, in AT, *n*-3 PUFAs are known to improve insulin sensitivity by increasing adiponectin secretion [38,39,40], enhancing glucose uptake through an increase in GLUT-4 content [41] and reducing leptin and ObR levels [42]. In addition, *n*-3 PUFAs have been shown to enhance adipogenesis and VEGFα release through PPARγ activation, which helps counteract hypoxia and reduce AT inflammation [43,44]. Accordingly, supplementation with EPA (1% of total energy intake) has been shown to preserve glucose homeostasis and reduce fat mass accumulation in mice fed an obesogenic diet, with a particular reduction in visceral AT mass [45,46]. However, the effects of *n*-3 PUFAs on weight loss are likely weak, as shown in a mouse model of diet-induced obesity, where no significant effects were observed, although accelerated mammary tumor growth was prevented [47].

Whereas transgenerational supplementation with EPA in mice has been shown to reduce whole body weight and metabolic disorders in HFD mice [48,49], clinical evidence that *n*-3 PUFAs may benefit weight loss or reduced fat mass in humans is less consistent, except when they are combined with calorie restriction [50].

A systematic review of randomized clinical trials found an association between *n*-3 PUFA intake and reduced plasma biomarkers of inflammation and endothelial activation, such as C-reactive protein, pro-inflammatory cytokines (IL-6, TNF-α), adhesion molecules (ICAM-1, VCAM-1), and eicosanoids, and suggested that *n*-3 PUFA supplementation may alleviate obesity-related inflammation [51]. Accordingly, a higher proportion of *n*-3 PUFAs in red blood cell (RBC) membranes has been associated with a lower fat mass index, reduced leptin levels, and decreased inflammatory markers such as C-reactive protein (CRP) and triglycerides [52,53,54].

Therefore, despite mixed findings on body weight, *n*-3 PUFAs may provide benefits with respect to inflammation and metabolic disorders in patients with unhealthy obesity [55].

### 2.2. Effects of n-3 PUFAs on Obesity-Related Metabolic Diseases

#### 2.2.1. Cardiovascular Disease

Cardiovascular disease (CVD) is one of the leading causes of death and morbidity worldwide. Obesity associated with chronic low-grade inflammation is an important risk factor for the development of metabolic CVD [56]. Consumption of *n*-3 PUFAs may confer benefits in the prevention of cardiovascular risk, such as dyslipidemia, inflammation, and blood pressure associated with obesity, but this is less evident at a therapeutic level. Indeed, through their antioxidant and anti-inflammatory effects, *n*-3 PUFAs have been shown to reduce autonomic sympathetic nerve activity in the hearts of rats, leading to the lowering of cardiovascular risks [57]. Supporting these results, DHA administration in rats has been shown to reduce heart rate and blood pressure, and thus cardiovascular risks, through activation of the FFA receptor in cardiac-projecting nucleus ambiguus neurons, which provide parasympathetic control of heart rate [58].

But, in humans, an extensive meta-analysis study of 86 clinical trials and 162,796 participants concluded that *n*-3 PUFAs, such as EPA or DHA, have little or no effect on reducing cardiovascular mortality and cardiovascular events, but slight effects in terms of reducing serum triglyceride, coronary heart disease (CHD) mortality, and CHD events. However, increasing ALA dietary intake from dried fruits or enriched margarine resulted in slightly reducing the risks of cardiovascular events and arrhythmia [59]. Overall, numerous prospective and meta-analytic studies agree that *n*-3 PUFA supplementation may substantially reduce the risks of CHD from 15 to 25%, depending on the study [60], with the exception of the ASCEND study, which was carried out on patients with T2D but without existing CVD at baseline. Indeed, the authors did not find any benefit in consuming 1 g/d of *n*-3 PUFAs as compared to olive oil, as there was no reduction in the primary end points of serious vascular events, such as ischemic attack, nonfatal myocardial infection, stroke, or vascular death. This was supported by the prospective VITAL study, which also used 1 g/d of fish oil capsule and found no benefit in humans without CVD at baseline [61]. At present, recommendations are in favor of a 1 g/d intake of *n*-3 PUFAs, but only in persons with prevalent CHD or heart failure [62].

Interventional studies in patients with or at risk of CVD showed less consistent effects when *n*-3 PUFAs were used as adjunctive therapy to statins. In support of the beneficial effects of *n*-3 PUFAs in reducing CVD outcomes is the REDUCE-IT clinical trial, which was carried out on patients with established cardiovascular disease, diabetes, or other CVD risk factors, such as hypertriglyceridemia, and who had been receiving statin therapy. They were treated with 3.6 g/d of EPA ethyl ester compound, which resulted in reducing the risks of the primary composite end points of cardiovascular death, nonfatal myocardial infarction, nonfatal stroke, coronary revascularization, or unstable angina by 25%, as compared to the placebo group who received mineral oil [63]. In the EVAPORATE study, by using the same treatment, at the same dose, in a high cardiovascular risk population treated with statins and with persistently high triglyceride levels, a beneficial effect was again found, as the progression of total (calcified and non-calcified) coronary plaque was slowed by 42% [64]. Disagreeing with these studies, in the STRENGTH study, in which both EPA and DHA were given (2.2 g and 0.8 g, respectively) to statin-treated patients with high cardiovascular risk, high triglycerides, and low HDL cholesterol levels, no effect on major cardiovascular events was observed, as compared to corn oil in the placebo group [65]. Moreover, such intake resulted in an increase in low-density lipoprotein (LDL) cholesterol levels even though high-density lipoprotein (HDL) cholesterol increased. This contributed to the decision to discontinue the trial, as a higher risk of atrial fibrillation was reported to occur when *n*-3 PUFA supplementation exceeded 1 g/day [66].

The reason for these discrepant results was attributed to the *n*-3 PUFA molecules used in these studies (EPA versus EPA + DHA), since EPA alone resulted in decreasing LDL cholesterol by 6.6%, whereas EPA + DHA increased it by 3%. The different placebos used (mineral oil versus corn oil) were also proposed as a possible explanation, as discussed by Djuricic et al. [60]. It seems also likely that environmental factors, such as lifestyle, dietary pattern, physical activity, and disease management, may have interfered with the efficiency of *n*-3 PUFAs with respect to CVD [67]. Moreover, there is evidence that statins may also exert antagonistic effects on *n*-3 PUFAs [68]. Indeed, while *n*-3 PUFAs are known to limit ischemia–reperfusion injury through improvement of mitochondrial function, one of the antagonistic effects of statins is the impairment of mitochondrial respiration [69]. *n*-3 PUFAs are also known to modulate excitability of myocytes, thus reducing arrythmias, but while these effects require the proper functioning of the cytochrome P450 (CYP) enzyme, statins may affect the production of eicosanoids through interactions with CYP [70,71]. Thus, the doses and the different conditionings of statins may also have played a role in these discrepant results.

In conclusion, the beneficial effects of *n*-3 PUFAs on CVD appear to be mainly observed at low doses of ≤1 g/day in the secondary prevention of CHD in patients with or at risk of CHD. Increasing the doses of *n*-3 PUFAs to more than 1 g/d, from an adjunctive therapeutic perspective, carries arisk of atrial fibrillation. Robust clinical trials are still needed to take into consideration the type (dietary or supplemental), dose, and duration of *n*-3 PUFA intervention, as well as lifestyle factors (sedentary or exercise) and possible synergy with other compounds that may interfere with *n*-3 PUFAs.

#### 2.2.2. Type 2 Diabetes (T2D)

T2D represents a major metabolic disorder most often related to obesity-mediated inflammation. Consumption of *n*-3 PUFAs may confer several benefits in terms of both the prevention and treatment of T2D, primarily through modulation of inflammation and insulin sensitivity [72]. *n*-3 PUFAs act on glucose metabolism through multiple interconnected pathways in T2D. At the cellular level, these PUFAs demonstrate remarkable influence on pancreatic β-cell function, enhancing their insulin secretion capacities while providing protection against oxidative damages. These effects are particularly achieved through improvement of the mitochondrial function and stimulation of insulin release through action on calcium channels [73]. Accordingly, a recent study has highlighted the role of *n*-3 PUFAs in improving mitochondrial biogenesis and function, particularly in insulin-sensitive tissues, resulting in better glucose utilization and reduced insulin resistance [74]. Upregulation of the nuclear factor erythroid 2-related factor 2 (Nrf2), a cellular antioxidant, by *n*-3 PUFAs also helps to alleviate T2D, as oxidative stress is a major contributor to β-cell dysfunction and insulin resistance [75]. Moreover, the influence of *n*-3 PUFAs extends beyond the pancreatic function to encompass broader metabolic regulation. A significant aspect of their action involves the modulation of glucose transport and insulin signaling in peripheral tissues. Notably, EPA has been shown to enhance glucose uptake through activation of the AMP-activated protein kinase (AMPK) pathway in muscles, leading to improved insulin-stimulated glucose disposal [76]. Of particular importance in T2D is the ability of *n*-3 PUFAs to modulate the incretin function, as demonstrated by their capacity to enhance glucagon-like peptide-1 (GLP-1) secretion and sensitivity, thereby improving both insulin secretion and glycemic control. This mechanism represents a distinct pathway by which *n*-3 PUFAs may influence glucose homeostasis, and suggests their use as an adjunct to other therapies in the management of T2D [77].

Numerous intervention studies have provided clinical validation of reductions in fasting blood glucose and insulin resistance, which was confirmed by a meta-analysis [78]. The therapeutic potential of *n*-3 PUFAs in T2D has been demonstrated in a comprehensive meta-analysis of 45 randomized controlled trials, in which *n*-3 PUFA supplementation has been shown to significantly induce hypolipidemic effects, together with a reduction in pro-inflammatory mediator and HbA1c levels [72].

#### 2.2.3. Metabolic Dysfunction-Associated Steatotic Liver Disease (MASLD)

MASLD, characterized by excessive fat accumulation and inflammation in the liver, is a major complication of obesity. As in the case of CVD and T2D, *n*-3 PUFAs have emerged as a possible adjunct therapy in the management of MASLD [79]. Molecular understanding of their effects in MASLD comes from the discovery of their ability to modulate liver inflammation. Indeed, Jump et al. demonstrated that *n*-3 PUFAs inhibit the NF-κB pathway in the liver, leading to decreased transcription of pro-inflammatory cytokines and reduced liver inflammation [80]. *n*-3 PUFAs have also demonstrated liver protective effects by reducing neutrophil infiltration and enhancing the clearance of apoptotic cells, a process particularly crucial in the resolution of liver inflammation [81]. In addition, *n*-3 PUFAs have also demonstrated a potent ability to modulate Toll-like receptor signaling in liver cells, with DHA reducing liver expression of the TLR2 and TLR4 receptors, as well as their co-receptor CD14, thus contributing to the anti-inflammatory effects of *n*-3 PUFAs in liver [82]. DHA has also been shown to inhibit NLRP3 inflammasome activation in the liver, thereby reducing inflammation and preventing the progression from simple steatosis to steatohepatitis [83]. Recent advances in this area have revealed significant effects of *n*-3 PUFAs on liver mitophagy, as DHA induce mitophagy of damaged mitochondria through activation of the GPR120/ERK pathway, thus leading to a reduction in mitochondrial dysfunction and oxidative stress [84]. The improved mitochondrial function in hepatocytes by *n*-3 PUFAs has been supported by a study in which DHA supplementation significantly reduced the levels of markers of oxidative stress, liver damage (plasma alanine [ALT] and aspartate [AST] amino-transferase), and inflammation [85,86]. Finally, maresin 1, a DHA-derived pro-resolving mediator, has been shown to act as a ligand for the nuclear receptor RORα, which is involved in the protection of liver inflammation through M2 polarization and in the attenuation of MASLD progression [87]. *n*-3 PUFAs also affect liver lipid metabolism by (i) down-regulating SREBP-1 transcription, thereby reducing de novo lipogenesis [88,89]; (ii) modifying the composition of very low-density lipoproteins (VLDL) with less palmitic, palmitoleic, and oleic acids; and (iii) reducing triglyceride secretion [90].

In support to these finding, the clinical validation of the *n*-3 PUFA effects on MASLD has been particularly well documented through several key interventional studies. The landmark WELCOME study (Wessex Evaluation of fatty Liver and Cardiovascular markers in NAFLD with OMacor thErapy), a randomized, placebo-controlled study, provided substantial evidence of the beneficial effects of *n*-3 PUFAs by showing a significant reduction in liver steatosis, which was positively correlated with DHA enrichment in red blood cells [91]. Moreover, recent long-term observational data from the UK Biobank have provided compelling evidence for the broader protective effects of *n*-3 PUFAs in liver disease. This comprehensive cohort study showed that a regular *n*-3 PUFA intake was associated with a 28% reduced risk of liver inflammatory disease, including alcoholic or non-alcoholic liver disease, and liver failure, while this was not the case with multivitamins, vitamin C, or vitamin B12 [92].

These studies have been further validated by recent meta-analyses. Thus, an umbrella review of 6561 participants demonstrated significant improvements in liver enzymes, with reductions in ALT (−6.72 IU/L), AST (−3.73 IU/L), and GGT levels (−4.20 IU/L), as well as liver fat content (−5.16%) [93]. A systematic review of studies from 2018–2023 confirmed these benefits, showing significant decreases in liver enzymes and improvements in serum lipid profiles [94]. These data were supported by a meta-analysis study which indicated an association of *n*-3 PUFA intake with improvement in metabolic parameters, liver enzyme levels, and steatosis score [95]. The integration of *n*-3 PUFAs into MASLD management strategies remains an active area of research, with emerging evidence in favor of their potential role in the prevention and treatment of obesity-related MASLD.

#### 2.2.4. Chronic Kidney Disease (CKD)

CKD represents another comorbidity of obesity-mediated inflammation, with significant morbidity and mortality worldwide. A significant advancement in understanding the mechanisms involved in the beneficial effects of *n*-3 PUFAs comes from the identification of specialized pro-resolving mediators (SPMs). Indeed, in the context of CKD, these compounds showed protective mechanisms able to mitigate renal inflammation and dysfunction, as assessed by a randomized controlled trial, in which *n*-3 PUFA supplementation increased the production of pro-resolving mediators, such as RvE1 and RvD5, which was correlated with improved inflammatory markers and renal function [96]. *n*-3 PUFAs may also improve renal hemodynamics, as shown by their ability to reduce the plasma levels of 20-hydroxyeicosatetraenoic acid (20-HETE), a potent vasoconstrictor, in patients with advanced stages of kidney disease. This was also associated with improved blood pressure control [97]. In addition, *n*-3 PUFAs have also shown significant antioxidant properties by reducing the levels of LDL and reactive-oxygen species (ROS) in end-stage renal disease hemodialysis patients [98].

Clinical evidence for the efficacy of *n*-3 PUFAs in CKD has been provided by several multiple intervention studies. A comprehensive meta-analysis of 17 clinical trials showed that *n*-3 PUFAs significantly reduced proteinuria in patients with CKD [99]. Moreover, PUFAs may also significantly reduce the serum levels of pro-inflammatory cytokines, such as TNF-α and IL-6, in hemodialysis patients [100]. Regarding cardiovascular outcomes, which are particularly important given the high cardiovascular burden in CKD patients, Svensson et al. found that *n*-3 PUFAs significantly reduced the risk of myocardial infarction in chronic hemodialysis patients [101]. A comprehensive meta-analysis also reported that PUFAs significantly reduced triglyceride levels and increased HDL cholesterol in hemodialysis patients [102].

In summary, *n*-3 PUFAs are likely to exert beneficial effects, from early CKD to hemodialysis patients.

### 2.3. n-3 PUFAs in Obesity Related-Chronic Inflammatory Immune Diseases

#### 2.3.1. Mechanisms of AT Low-Grade Inflammation in Obesity

Under physiological conditions, AT is infiltrated by anti-inflammatory immune cells, including M2 macrophages, regulatory T cells that secrete IL-10, and type 2 innate lymphoid cells. However, during obesity development, adipocyte hypertrophy limits oxygen supply to adipose tissue, inducing hypoxia [103]. This hypoxic state leads to the overexpression of hypoxia-inducible factor 1-alpha (HIF1α) by mature adipocytes and adipocyte precursors, promoting neovascularization [104]. Additionally, hypoxia induced secretion of monocyte chemoattractant protein-1 (MCP-1) and CCL5 by adipose tissue-derived stem cells (ASCs), contributing to the recruitment of immune cells, including circulating lymphocytes and monocytes [105,106,107]. AT is then infiltrated by pro-inflammatory immune cells, such as CD4+ (Th1) T cells, CD8+ T cells, and M1 macrophages. CD8+ T cells, which are the first cells to infiltrate AT, play a role in the recruitment and activation of type 1 macrophages. Indeed, gene depletion of CD8+ T cells has been shown to reduce macrophage recruitment and AT inflammation [108]. These processes lead to the development of a pro-inflammatory environment within obese AT, characterized by the presence of pro-inflammatory cytokines such as TNFα [109,110], IL1β, IL8, and IL-6, increased levels of leptin, and decreased levels of adiponectin [111], which are implicated in the disruption of insulin signaling. In addition, we have demonstrated the leading role of ASCs in promoting Th17 cell polarization and reducing insulin sensitivity in adipocytes [112,113]. Accordingly, obesity has been shown to promote expansion of Th17 cells in AT or periphery in a diet-induced obesity (DIO) murine model [114] as well as in humans. Indeed, infiltrating IL-17+-secreting cells have been found to be more abundant in obese AT than lean AT, and in visceral rather than subcutaneous AT [115]. Moreover, significant increases in circulating IL-17 and IL-23 cytokines were observed in obese individuals as compared to lean individuals [116]. Finally, Dalmas et al. have shown that several sub-populations of CD4+ cells secreting IL17, together or not with IL-22, were highly induced in the AT of obese subjects as compared to non-obese subjects [116]. Through binding to its ubiquitous receptors, IL-17 plays a crucial role in the propagation of inflammation, leading to the development of a low-grade chronic inflammation within AT and contributing to chronic inflammatory diseases associated with obesity.

#### 2.3.2. Shared Mechanisms of Inflammation in Obesity and Related Chronic Inflammatory Diseases

Chronic inflammation within AT promotes an inflammatory microenvironment with increased expression of soluble factors such as adipokines, cytokines, metalloproteinases, pro-angiogenic factors, and ROS [117]. Through propagation of sustained inflammation, obesity thus exacerbates several chronic inflammatory diseases [118]. Indeed, increased adiposity is associated with a worse outcome in terms of autoimmune diseases such as psoriasis, inflammatory bowel disease (IBD), and multiple sclerosis (MS) [119]. Obesity-induced inflammation also aggravates rheumatic diseases through enhanced production of pro-inflammatory mediators and altered immune responses [120,121,122]. Additionally, recent evidence suggests that obesity-related inflammation may accelerate the progression of neurodegenerative diseases, including Alzheimer and Parkinson’s diseases [116]. Furthermore, obesity has been associated with increased cancer risk and poorer prognosis, in part due to the pro-inflammatory environment that promotes tumor growth and progression, and to the contribution of ASCs in upregulating immune check point expression in adipocytes and neighboring cancer cells [4,123,124].

**Figure 2:**
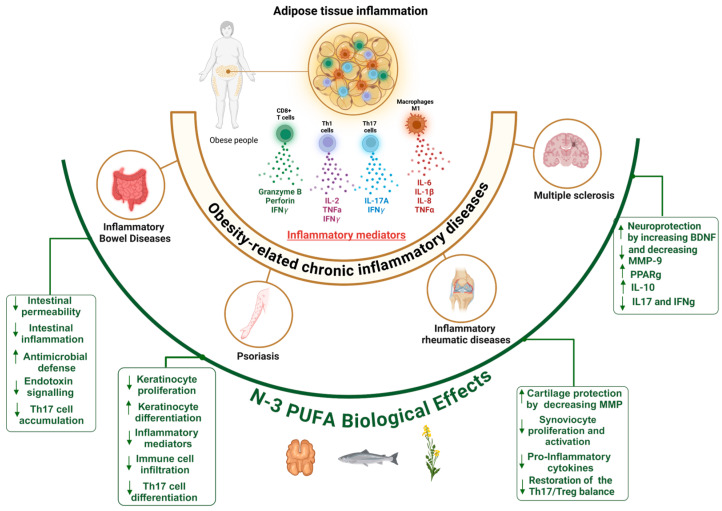
Biological effects of *n*-3 PUFAs on chronic inflammatory diseases related to obesity. Obesity exacerbates several chronic inflammatory immune diseases due to its low-grade inflammatory state. *n*-3 PUFAs contribute to the resolution of such inflammatory diseases though multiple pathways. Up and down arrows correspond to enhancing or inhibiting effects, respectively.

In the past, a subset of Th17 lymphocytes, the Th17.1 lymphocytes, has been identified in several inflammatory and auto-immune diseases such as inflammatory bowel disease (IBD), multiple sclerosis (MS), psoriasis, and rheumatoid arthritis (RA), which are all aggravated by obesity. The Th17.1 lymphocyte subset presents the particularity of secreting both IL-17A and IFN-γ (a signature cytokine for Th1 lymphocytes) [125,126]. One of the key drivers leading to Th17.1 generation is the cytokine IL-23, as IL-6 + TGFβ without IL-23 leads to the generation of physiological Th17 cells, whereas the addition of IL-23 leads to Th17.1 cell generation [127,128]. In addition to pathogenic Th17.1 cell driving, IL-23 restrains the activity of regulatory T cells [129]. Interestingly, a dysregulation of the Th17/Treg balance has been observed in obesity [130]. Furthermore, we have shown that Th17.1 cells are promoted by ASCs in AT from obese, but not lean, individuals [112]. Moreover, we have also reported the ability of *n*-3 PUFAs to decrease ASC-mediated Th17-cell activation by reducing ICAM-1 expression [131]. In agreement with this, *n*-3 PUFAs have been shown to reduce membrane anchoring of IL-6 receptors on naïve lymphocytes, decreasing their polarization into Th17 lymphocytes [132]. Taken together, these studies suggest that *n*-3 PUFAs may alleviate obesity-related chronic inflammatory diseases through multiple mechanisms, including a reduction in IL-17 A secretion and subsequent propagation of inflammation, as shown in Figure 2.

These mechanisms highlight the broad impact of obesity-mediated AT inflammation on related inflammatory diseases. Their effects will be discussed herein, with the exception of cancer.

#### 2.3.3. Inflammatory Bowel Diseases (IBDs)

Inflammatory Bowel Diseases (IBDs), including Crohn’s disease and ulcerative colitis, are chronic inflammatory diseases closely linked to obesity-mediated inflammation. The chronic low-grade inflammation associated with obesity creates a fertile ground for intestinal inflammatory processes, where immune dysregulation and metabolic disruptions converge [133]. Epidemiological links have been reported between obesity and IBD [134]. Excess AT generates pro-inflammatory cytokines that can exacerbate intestinal inflammation, while simultaneously compromising the gut microbiome’s delicate balance [135]. Therapeutic approaches targeting inflammatory pathways have gained increasing attention. Among these, *n*-3 PUFAs have emerged as promising agents, offering multiple mechanisms to address both metabolic and inflammatory aspects of IBD [136]. Using a mouse model of HFD + trinitrobenzene sulfonic acid (TNBS)-induced colitis, dietary *n*-3 PUFAs have been shown to reduce colitis-associated disease severity and colonic mRNA expression of cytokines (IL-6, IL-17A, IL-17F, IL-21, IL-23, and IFNγ) versus corn oil in control HFD mice. Concomitantly, adipose tissue mRNA expression of inflammatory cytokines (MCP-1, IFNγ, IL-6, IL17F, and IL-21) and macrophage infiltration were reduced [137]. This was supported using the Fat-1 mouse model, which constitutively produces *n*-3 PUFAs and in which chronic colitis was induced by dextran–sodium–sulfate (DSS) during 3 cycles of 5 days. Indeed, colon histological scores were improved, together with reduced spleen and colonic mucosal IL-17 and CCR6 mRNA levels, compared to WT mice [138]. In addition to the ability of *n*-3 PUFAs to modulate inflammatory pathways, Willemsen et al., who investigated the effects of PUFAs on epithelial barrier integrity, reported that DHA, AA, dihomo-gamma-linolenic acid (DGLA), and EPA increase basal transepithelial resistance and significantly reduce IL-4-mediated permeability in human intestinal epithelial cells [139]. In support to this study, DHA limited the effect of inflammatory stimulus on occludin, zona occludins-1, and barrier function when Caco-2 cells were exposed for 24 h to a cocktail of pro-inflammatory cytokines (IL-1β/TNF-α/IFNγ) at their basolateral side and to LPS at both sides [140]. Interestingly, even without cell injury, the addition of EPA enhanced permeability and transepithelial resistance in Caco-2 confluent cells, as compared to the addition of AA or LA, or even oleic acid (OA) [141]. These observations suggest that *n*-3 PUFAs may maintain the intestinal barrier function and combat inflammation-mediated permeability. Beyond these direct effects on the intestinal barrier function, SPMs have been reported to be a promising innovative approach to the treatment of IBD, as they contribute to the resolution of inflammation in IBD through binding to G protein coupled receptors [142]. They have also been shown to reduce neutrophil infiltration, stimulate phagocytosis and efferocytosis, enhance antimicrobial defense, attenuate endotoxin signaling, reduce mucosal pro-inflammatory cytokine production, and improve wound repair [142,143].

However, the clinical evidence for the use of *n*-3 PUFAs in the treatment of IBD is mixed. In ulcerative colitis disease (UC), a meta-analysis was inconclusive on the benefits of *n*-3 PUFA supplementation with respect to increasing disease remission [144], which was confirmed in a more recent review [145]. Nevertheless, a modest reduction in the need for corticosteroids in patients with active disease and a delay of early relapse via *n*-3 fatty acids have been reported in UC [146,147]. However, gastrointestinal side effects such as diarrhea and upper digestive symptoms have been reported in patients receiving *n*-3 PUFA supplementation [144]. In Crohn’s disease, statistically significant benefits have been reported, especially in children where a randomized controlled trial showed significantly lower relapse rates at one year when receiving enteric-coated *n*-3 PUFA supplementation compared to a placebo (61% vs. 95%, *p* = 0.0016) [148]. Finally, a recent study reported that a Mediterranean diet may reduce active disease and inflammatory markers in both UC and Crohn’s disease due to its high content of biologically active foods, including nuts and fish, which are rich in *n*-3 PUFAs [149].

While adult studies have shown limited efficacy and side effects, pediatric data with enteric-coated formulations have demonstrated more encouraging results. This divergence suggests that factors such as age or formulation may be important determinants of success. However, until further clinical trials are concluded, a Mediterranean diet should be proposed as an additive strategy in the treatment of IBD.

#### 2.3.4. Psoriasis

Psoriasis represents a chronic inflammatory skin disease which is impacted by obesity-mediated inflammation. Like other obesity-related disorders, psoriasis demonstrates complex interactions between metabolic dysfunction and immune dysregulation. Recent epidemiological studies have established strong associations between obesity and psoriasis onset or severity, as elevated BMI is positively correlated with increased disease burden [150]. Thus, obesity acts as an independent risk factor for the development and worsening of psoriasis [151]. Obesity and psoriasis exhibit similar systemic inflammatory profiles, with elevated serum levels of cytokines such as TNF-α, IL-6, and IL-12 [152]. Interestingly, weight loss has been shown to have a beneficial effect on psoriasis severity and treatment response [153]. Tissue-engineered skin models used to investigate the impact of *n*-3 PUFAs on psoriasis demonstrated that supplementation in a culture medium with 10 μM ALA results in the incorporation of its EPA metabolite into the epidermal phospholipid fraction, inhibition of keratinocyte proliferation, and restoration of keratinocyte differentiation capacity, and thus a decrease in the pathological phenotype of psoriatic skin substitutes [154]. Likewise, DHA supplementation mitigates psoriatic characteristics by normalizing epidermal cell differentiation and proliferation and by decreasing the levels of lipid mediators derived from *n*-6 PUFAs, mainly prostaglandin E2 (PGE2) and 12-hydroxyeicosatetraenoic acid (12-HETE), in a 3D tissue-engineered skin model [155].

As mentioned above, psoriasis is a chronic inflammatory skin disease induced via the IL-23/IL-17 axis. While SFAs can exacerbate psoriatic dermatitis through activation of the inflammasome in keratinocytes and induction of IL-17-producing cells such as Th17 and IL-17-producing γδ T cells in the skin, *n*-3 PUFAs may, on the contrary, suppress Th17 differentiation [156]. Accordingly, in a 3D psoriatic skin model, EPA normalized the proliferation of psoriatic keratinocytes and diminished the levels of IL-17 [157]. Supporting these results, activation of the GPR120/FFAR4 receptor with agonists in a psoriasis model resulted in the attenuation of skin lesions, a decrease in inflammatory cytokine levels, and inhibition of Th17 cell differentiation [158]. Accordingly, topical treatment with 30 μM DHA in rats was shown to accelerate wound healing and reduce inflammatory mediator production through GPR120 activation in skin [159].

Significant progress has also been made on the identification of SPMs derived from *n*-3 PUFAs in psoriatic skin. Indeed, resolvin E1 (RvE1) has been demonstrated to reduce inflammatory cell infiltration and epidermal hyperplasia in a murine model of psoriasis and to decrease IL-23 expression and production [160]. Similarly, resolvin D1 (RvD1) and resolvin D5 (RvD5) have been shown to exert anti-inflammatory effects by reducing the expression of interleukin-24 and S100A12 in human keratinocytes [161]. In dietary interventions using a mouse model of psoriasis, DHA showed more pronounced effects than EPA in reducing inflammation by increasing skin RvD5, protectin D, and maresin 2 levels [162].

The clinical validation of *n*-3 PUFAs in the treatment of psoriasis is provided by the analysis of multiple interventional studies. In a systematic review of 18 randomized controlled trials, it was concluded that supplementation with *n*-3 PUFAs, but only when they were combined with conventional treatments, decreases several inflammatory mediators and reduces psoriasis severity, as quantified by the Psoriasis Area Severity Index (PASI) score and lesion area. In addition, *n*-3 PUFAs were also found to reduce the risks of obesity and cardiovascular and metabolic diseases [163]. Another meta-analysis provided robust evidence supporting the efficacy of *n*-3 PUFA supplementation in the amelioration of several psoriasis parameters. Specifically, a dose-dependent improvement in clinical manifestations, including erythema, scaling, and pruritus, was seen with *n*-3 PUFA supplementation [164]. Thus, *n*-3 PUFAs and their derivatives are likely to become a promising adjunct approach for the treatment of psoriasis.

#### 2.3.5. Inflammatory Rheumatic Diseases

##### Osteoarthritis

Rheumatic diseases, particularly osteoarthritis (OA) and, to a lesser extent, rheumatoid arthritis (RA), are associated with obesity. The relationship between obesity and OA extends beyond mechanical stress, with obesity being identified as one of the main risk factors contributing to both incidence and progression [165,166], with a critical role in terms of systemic and chronic low-grade inflammation [167]. AT acts as an endocrine organ through the release of soluble mediators, including cytokines, adipokines, and ROS, which induce detrimental effects on joint tissues [168]. Most adipokines (leptin, visfatin, and resistin) promote the activation of an inflammatory circuit, known as meta-inflammation, leading to the synthesis of degradative enzymes, ROS, and prostaglandins [117,168]. But IL-1β is one of the most active cytokines that leads to cartilage destruction, due to its ability to activate MMP 1 and MMP 13, which results in the loss of sulphated glycosaminoglycans (sGAG) and type II collagen [169]. Using an in vitro model of equine IL-1β-synoviocyte culture, Caron et al. demonstrated that the addition of DHA to cultures resulted in the incorporation of DHA in synoviocytes, which was associated with a reduction in MMPs, IL1β, IL-6, and Cox-2 expression. Moreover, DHA-derived SPMs, RvD1, RvD2, Maresin1, and Protectin DX were also able to abrogate MMP-1, MMP-13, and IL-6 gene expression in IL-1β-stimulated synoviocytes [170]. Supporting the beneficial effects of *n*-3 PUFAs on IL-1β-mediated cartilage degradation, Wann et al. demonstrated that the addition of EPA or DHA at a 0.1 or 1 μM concentration resulted in less IL-1β-mediated sGAG loss [169]. Moreover, in a high-fat-diet mouse model where cartilage degradation was surgically induced, *n*-3 PUFA, but not n6 PUFA or SFA, supplementation resulted in the inhibition of TLR4/NF-κB and NLRP3/caspase-1 expression and signaling, which led to a reduction in inflammatory response and IL-1β-mediated pyroptosis in chondrocytes [171]. Accordingly, another study, also using a high-fat-diet mouse model, demonstrated that supplementation with *n*-3 PUFAs, but not with *n*-6 PUFAs, attenuated injury-induced OA, decreased leptin and resistin levels, and significantly enhanced wound repair [172].

In humans, several clinical trials have demonstrated the positive effects of *n*-3 PUFAs in terms of managing OA. A comprehensive meta-analysis of nine randomized controlled trials involving 2070 patients with OA concluded that *n*-3 PUFA supplementation significantly alleviates arthritis pain and improves joint function compared to a placebo [173]. However, another study, using genome-wide association study (GWAS) and Mendelian randomization (MR) methods, concluded that PUFA may not be helpful in OA [174]. But, *n*-3 PUFAs, coupled with emerging optimized delivery methods, particularly through liposomal formulations, should offer new perspectives for improving their therapeutic efficacy, as demonstrated by the success of liposome-encapsulated RvD1 delivery in the joints of obese mice with OA [175].

##### Rheumatoid Arthritis

In rheumatoid arthritis (RA), the link with obesity is not as high as in OA. Indeed, the odds ratio of RA risk is 1.32 in patients with obesity [176], while it reaches 4.55 for knee OA [177], as calculated in a meta-analysis, and approximately 2 for hip OA [178]. However, obesity appears to be a clear risk factor for a poor response to treatment, regardless of whether a biologic or non-biologic therapy is being used [120,179,180].

Th17 cells play a central role in RA by inducing synoviocyte inflammation and neutrophil and macrophage infiltration [181]. In this context, we have previously shown the preponderant role of mesenchymal stem cells and synoviocytes in promoting Th17 cell polarization [182], a mechanism that is closely similar to the one we have demonstrated in obesity with ASCs and adipocytes [112,113]. Using the Fat-1 mouse model, which endogenously produces *n*-3 PUFAs, increased *n*-3 PUFA levels have been shown to attenuate inflammatory arthritis. This effect was associated with reduced IL-17 production and increased FoxP3 expression, therefore promoting Treg differentiation [183]. A similar finding was reported in a model of collagen-induced arthritis, where activation of the GPR120/FFAR resulted in restoring the Th1/Th17 and Treg balance [184]. *n*-3 PUFAs have also been reported to decrease antigen presentation via MHC II, inflammatory cytokine production by monocyte/macrophages, and ROS production by various leukocytes in RA [185].

In clinical intervention studies, a systematic review has found an inverse association between RA risk and oily fish consumption, even though this association was not linear [186]. Supporting these results, combining *n*-3 PUFA supplementation with an anti-inflammatory dietary intervention that provides an arachidonic acid intake of less than 90 mg/day has demonstrated significant reductions in CRP, TNF-α, number of tender joints, LTB4 levels, and the dose of corticosteroids used [187]. In a meta-analysis study, consumption of *n*-3 PUFAs has also been found to significantly improve disease-activity-related markers and reduce blood leukotriene B4 and triacylglycerol levels [188]. Finally, the benefits of *n*-3 PUFAs in terms of reducing (i) duration of morning stiffness, (ii) number of tender or swollen joints, (iii) time to fatigue, (iv) pain or disease activity, and (v) use of NSAIDs and increasing grip strength have been reported in RA [185].

In conclusion, these findings collectively support the therapeutic potential of *n*-3 PUFAs for RA, while highlighting the importance of limiting saturated and *n*-6 PUFA intake.

#### 2.3.6. Multiple Sclerosis

Multiple sclerosis (MS) is an autoimmune disease characterized by inflammation and damage to axons leading to demyelination and formation of plaques in the central nervous system (CNS) [183]. Obesity is known to influence the progression and severity of this disease [189].

The effects of *n*-3 PUFAs on MS have been linked to their ability to regulate inflammation in the periphery and the central nervous system as well. DHA and EPA have indeed been found to cross the blood–brain barrier and directly impair neuroinflammatory processes [190]. Brain phospholipids enrichment in DHA more than EPA affects cell signaling by modifying the composition of lipid rafts [191]. *n*-3 PUFAs may also decrease the activity and production of matrix metalloproteinase-9, which is implicated in blood–brain barrier breakdown in MS, thereby preserving blood–brain barrier integrity [192]. In addition, *n*-3 PUFAs, especially DHA, demonstrate neuroprotective properties by promoting neuronal survival and neurite outgrowth [191]. Thus, *n*-3 PUFAs, such as DHA and EPA, have been shown to upregulate the expression of brain-derived neurotrophic factor (BDNF), which plays a role in promoting neuronal survival and synaptic plasticity [193,194]. In addition, SPMs derived from DHA, more particularly RvD1 and neuroprotectin D1, have also demonstrated neuroprotective properties [195].

Th17 cells, especially pathogenic Th17.1 cells, have been involved in MS physiopathology using the Experimental Autoimmune Encephalomyelitis model [196,197]. Of interest, *n*-3 PUFAs have demonstrated an ability to inhibit both IL-17 and IFNγ production in MS pathology, together with activation of PPARγ, but whether this results in inhibiting Th17.1 cell activation has not been demonstrated [198].

Clinical evidence on the efficacy of *n*-3 PUFA supplementation in the treatment of MS is mixed. Indeed, some studies have shown promising results, with a significant reduction in the mean annual exacerbation rate and the mean Expanded Disability Status Scale (EDSS) in newly diagnosed MS patients supplemented with fish oil, vitamins, and dietary advice [199], or a reduction in relapse rates and inflammatory markers and an improved quality of life in MS patients [200]. But this was not supported by other studies, although in one study *n*-3 PUFA supplementation (4 g/d, 12 months) resulted in a reduction in serum pro-inflammatory cytokines such as TNF-α, IL-1β, and IL-6 while increasing anti-inflammatory IL-10 levels in treated MS patients [201]. In another study, supplementation involved capsules with 1350 mg of EPA and 850 mg of DHA daily for 24 months, but no significant effect from *n*-3 PUFAs on disease activity or relapse rates was observed [202]. However, to reconcile these studies, Farinotti et al. reported that although polyunsaturated *n*-3 PUFAs do not appear to have a major effect on the main clinical outcome in MS, namely disease progression, they may tend to reduce the frequency of relapses over two years [203].

Thus, in light of these conflicting findings, it seems that more research is needed to fully understand the potential benefits of *n*-3 PUFA supplementation in the management of MS. Clinical trials combining neuroimaging biomarkers with cerebrospinal fluid analysis could help determine whether *n*-3 PUFAs exert direct neuroprotective effects beyond their anti-inflammatory action, potentially establishing them as adjunctive therapy for MS patients.

## 3. Conclusions

Our comprehensive review highlights the potential of *n*-3 PUFAs to contribute to the resolution of inflammation in obesity and related metabolic and chronic inflammatory diseases. These beneficial effects occur through several interrelated pathways, including activation of the GPR120/FFAR4 and PPARγ receptors and generation of anti-inflammatory eicosanoids and SPMs. In addition, *n*-3 PUFAs also demonstrate significant effects on T cell activation, specifically inhibition of Th1 and Th17 cell differentiation or activation, but promotion of regulatory T cell responses [132,183,184,204]. This immunomodulatory capacity extends to macrophages, as they can switch their balance from M1 to M2 macrophages and modulate their antigen-presenting cell function [205,206].

However, clinical evidence shows variable efficacy in the diseases that we have mentioned herein.

### 3.1. Limitations of Current Evidence

A comprehensive examination of *n*-3 PUFA research across obesity-related disorders highlights several significant limitations. Firstly, there exists an imbalance between preclinical and clinical evidence, with excessive reliance on murine models and insufficient translation to human applications. While mouse studies demonstrate robust effects of *n*-3 PUFA supplementation on fat mass prevention in high-fat-diet conditions, human trials show more modest outcomes, typically requiring a combination with caloric restriction to achieve significant weight loss.

In CVD research, inconsistent findings from major clinical trials raise methodological concerns. The REDUCE-IT study demonstrated cardiovascular benefits with high-dose EPA, whereas the STRENGTH trial using combined EPA + DHA showed no significant improvements. Such discrepancies may stem from differences in *n*-3 PUFA formulations, dosages, or control substances used for comparison.

Regarding T2D, despite promising mechanistic findings on insulin sensitivity and beta-cell function, clinical studies reveal variable glycemic improvements that do not consistently translate to meaningful HbA1c reductions across different patient populations. Similarly, in MASLD investigations, the WELCOME trial and subsequent studies demonstrate improvements in hepatic steatosis but provide limited data on histological outcomes or disease progression markers.

For inflammatory conditions, notable inconsistencies emerge across disease states. In inflammatory bowel disease, enteric-coated *n*-3 PUFA formulations show promising results in pediatric populations but limited efficacy in adults. Psoriasis studies similarly demonstrate considerable variability in clinical response, potentially attributable to differences in disease severity or concurrent conventional therapies.

Furthermore, methodological heterogeneity across studies complicates interpretation, including variations in *n*-3 PUFA sources, dosages (ranging from <1 g/day to >4 g/day), treatment duration, and administration routes. Moreover, the relationship between *n*-3 PUFAs and gender has rarely been investigated, despite their potential relevance, particularly in autoimmune conditions with known gender disparities.

Bioavailability issues represent another limitation, as conventional *n*-3 PUFA formulations may yield suboptimal tissue incorporation compared to emerging delivery systems such as liposomal preparations, which have shown superior results in preclinical models of rheumatic diseases. Additionally, while growing evidence suggests that gut microbiota mediates some *n*-3 PUFA effects, few studies adequately assess baseline microbiome composition or monitor changes throughout the intervention period.

These limitations underscore the need for well-designed clinical trials with appropriate patient stratification, extended follow-up periods, standardized dosing protocols, and clinically relevant endpoints to better define optimal therapeutic strategies for *n*-3 PUFA interventions in obesity-related inflammatory conditions.

### 3.2. Perspectives

However, there is evidence to suggest that earlier intervention may offer greater benefit in terms of preventing rather than treating the disease. SPMs derived from *n*-3 PUFAs represent a particularly promising area of investigation, especially when encapsulated into liposomes, which may offer a new therapeutic approach, notably in the field of obesity and its inflammatory comorbidities. The identification of new agonists, mimetics, and improved SPM formulations represents a promising approach to specifically target deficient resolution processes [207]. Moreover, the ability of SPMs to modulate adaptive immune responses, particularly on B and T lymphocyte populations, underscores their potential in treating autoimmune and chronic inflammatory diseases associated with obesity [208]. These therapeutic innovations, combined with a better understanding of the specific mechanisms of action of different SPMs, could enable more targeted and personalized approaches for patients suffering from obesity and its inflammatory complications.

## Abbreviations

The following abbreviations are used in this manuscript:

AA	Arachidonic acid	MS	Multiple Sclerosis
ALA	Alpha-linolenic acid	MUFA	Monounsaturated Fatty Acid
ALT	Alanine amino-transferase	MyD88	Myeloid Differentiation primary response 88
AMPK	AMP-activated protein kinase	*n*-3	Omega-3
AP-1	Activator Protein 1	*n*-6	Omega-6
ASC	Adipose-derived stem cells	NF-κB	Nuclear factor kappa B
AST	Aspartate amino-transferase	NLRP3	NOD-like receptor family pyrin domain containing 3
AT	Adipose tissue	Nrf2	Nuclear factor erythroid 2-related factor 2
BDNF	Brain-derived neurotrophic factor	OA	Osteoarthritis
BMI	Body mass index	ObOA	Obesity-associated osteoarthritis
CKD	Chronic kidney disease	PASI	Psoriasis Area Severity Index
COX	Cyclooxygenase	PD-L1	Programmed Death-Ligand 1
CRP	C-reactive protein	PDX	Protectin DX
CVD	Cardiovascular disease	PG	Prostaglandin
CYP	Cytochrome P450	PLA2	Phospholipase A2
DHA	Docosahexaenoic acid	PPAR	Peroxisome proliferator-activated receptor
DGLA	Dihomo-gamma-linolenic acid	PPRE	Peroxisome proliferator-activated receptor response element
EDSS	Expanded Disability Status Scale	PUFA	Polyunsaturated fatty acid
EPA	Eicosapentaenoic acid	RA	Rheumatoid arthritis
ERK	Extracellular signal-regulated kinase	RAGE	Receptor for advanced glycation end-products
ESRD	End-ctage renal disease	RBC	Red blood cell
FA	Fatty acid	RORα	RAR-related Orphan Receptor alpha
FFAR4	Free Fatty Acid Receptor 4	ROS	Reactive Oxygen Species
GLP-1	Glucagon-like peptide-1	Rv	Resolvin
GLUT-4	Glucose Transporter Type 4	RXR	Retinoid X receptor
GM-CSF	Granulocyte-Macrophage Colony-Stimulating Factor	SFA	Saturated fatty acid
GPR120	G protein-coupled receptor 120	SIRT1	Sirtuin 1
GSDMD	Gasdermin D	SPM	Specialized pro-resolving mediator
HbA1c	Hemoglobin A1c	SPMs	Specialized pro-resolving mediators
HDL	High-density lipoprotein	SREBP-1	Sterol Regulatory Element-Binding Protein-1
HIF1α	Hypoxia-inducible factor 1-alpha	T2D	Type 2 diabetes
HMGB1	High Mobility Group Box 1	TAB1	TAK1-binding protein 1
IBD	Inflammatory bowel disease	TAK1	Transforming growth factor beta-activated kinase 1
ICAM-1	Intercellular Adhesion Molecule 1	TGFβ	Transforming growth factor beta
IFN	Interferon	Th1	T helper 1
IKK-β	Inhibitor of Nuclear Factor Kappa-B	Th17	T helper 17
IL-	Interleukin-	TLR	Toll-like receptor
JNK	c-Jun N-terminal kinase	Treg	Regulatory T cell
LA	Linoleic acid	TXA	Thromboxane A
LDL	Low-density lipoprotein	UC	Ulcerative colitis
LOX	Lipoxygenase	VEGF	Vascular Endothelial Growth Factor
LPS	Lipopolysaccharide	VLDL	Very low-density lipoproteins
LTB4	Leukotriene B4		
LTB5	Leukotriene B5		
MASLD	Metabolic dysfunction-associated steatotic liver disease		
MCP-1	Monocyte chemoattractant protein-1		
MMP	Matrix metalloproteinase		
MRI	Magnetic resonance imaging		
